# Obligatory Role of Intraluminal O_2_^−^ in Acute Endothelin-1 and Angiotensin II Signaling to Mediate Endothelial Dysfunction and MAPK Activation in Guinea-Pig Hearts

**DOI:** 10.3390/ijms151119417

**Published:** 2014-10-27

**Authors:** Emilia Wojtera, Anna Konior, Natalia Fedoryszak-Kuśka, Andrzej Beręsewicz

**Affiliations:** Department of Clinical Physiology, Postgraduate Medical School, Warsaw 01-813, Poland; E-Mails: emiliak567@gmail.com (E.W.); niakon@wp.pl (A.K.); fedna@cmkp.edu.pl (N.F.-K.)

**Keywords:** oxidative stress, endothelin-1, angiotensin-II, endothelial dysfunction, mitogen-activated protein kinases, mitochondria, NADPH-oxidase, xanthine oxidase, guinea-pig heart

## Abstract

We hypothesized that, due to a cross-talk between cytoplasmic O_2_^−^-sources and intraluminally expressed xanthine oxidase (XO), intraluminal O_2_^−^ is instrumental in mediating intraluminal (endothelial dysfunction) and cytosolic (p38 and ERK1/2 MAPKs phosphorylation) manifestations of vascular oxidative stress induced by endothelin-1 (ET-1) and angiotensin II (AT-II). Isolated guinea-pig hearts were subjected to 10-min agonist perfusion causing a burst of an intraluminal O_2_^−^. ET-1 antagonist, tezosentan, attenuated AT-II-mediated O_2_^−^, indicating its partial ET-1 mediation. ET-1 and Ang-T (AT-II + tezosentan) triggered intraluminal O_2_^−^, endothelial dysfunction, MAPKs and p47phox phosphorylation, and NADPH oxidase (Nox) and XO activation. These effects were: (i) prevented by blocking PKC (chelerythrine), Nox (apocynin), mitochondrial ATP-dependent K^+^ channel (5-HD), complex II (TTFA), and XO (allopurinol); (ii) mimicked by the activation of Nox (NADH); and mitochondria (diazoxide, 3-NPA) and (iii) the effects by NADH were prevented by 5-HD, TTFA and chelerythrine, and those by diazoxide and 3-NPA by apocynin and chelerythrine, suggesting that the agonists coactivate Nox and mitochondria, which further amplify their activity via PKC. The effects by ET-1, Ang-T, NADH, diazoxide, and 3-NPA were opposed by blocking intraluminal O_2_^−^ (SOD) and XO, and were mimicked by XO activation (hypoxanthine). Apocynin, TTFA, chelerythrine, and SOD opposed the effects by hypoxanthine. In conclusion, oxidative stress by agonists involves cellular inside-out and outside-in signaling in which Nox-mitochondria-PKC system and XO mutually maintain their activities via the intraluminal O_2_^−^.

## 1. Introduction

Oxidative stress and endothelial dysfunction play a critical role in the pathogenesis of cardiovascular disease [1,2,3] and such states as post-ischemic inflammation and no-reflow phenomenon [4,5,6]. It is believed that cardiovascular risk factors, which function via such agents as angiotensin II (AT-II) and endothelin-1 (ET-1) [7,8,9], and ischemia/reperfusion acting via ET-1 [4,6,10] mediate the production of excess vascular reactive oxygen species (ROS), including superoxide (O_2_^−^). ROS, *per se*, or via the reduced nitric oxide (NO) bioavailability (seen as endothelial dysfunction) activate intracellular signaling molecules, such as mitogen-activated protein kinases (MAPKs) and transcription factors [2,8,11], which ultimately mediate vascular inflammation and remodeling. However, using antioxidants to prevent cardiovascular diseases and ischemia/reperfusion injury has been demonstrated to be ineffective in clinical trials [12,13] and most likely reflect our incomplete understanding of oxidative stress.

**Scheme 1:**
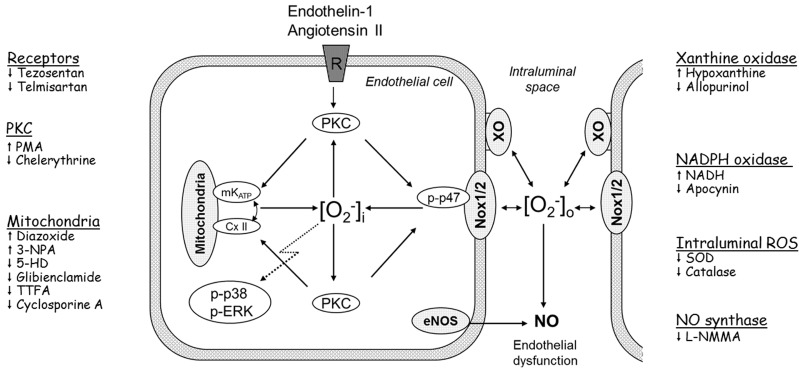
Proposed model of acute ET-1 and Ang II signaling to induce O_2_^−^, p38 and 1/2ERK (MAPKs) phosphorylation, and endothelial dysfunction. We propose that agonist-mediated signaling involves three steps: (i) The membrane receptor-protein kinase C (PKC)-dependent coactivation of NADPH oxidases (Nox, including Nox1/2 activation involving p47phox phosphorylation) and mitochondrial ATP-dependent K^+^ channel (mK_ATP_) and complex II (CxII) to generate O_2_^−^; (ii) A cross-talk Nox-mitochondria-PKC. Nox- and mitochondria-derived O_2_^−^ activates PKC (the same or its other pool), which further activates mitochondria and Nox, thereby controlling cytosolic O_2_^−^ and MAPKs activity; (iii) A cross-talk between the Nox-mitochondria-PKC system and xanthine oxidase (XO). Nox1/2-derived O_2_^−^ is partially released intraluminally to activate XO, and the O_2_^−^ from Nox1/2 and XO inactivates NO to mediate endothelial dysfunction. This O_2_^−^ also feeds-back to Nox1/2 to maintain the activity of the Nox-mitochondria-PKC system. The cytosolic and the intraluminal O_2_^−^-pool remain in equilibrium, likely due to Nox1/2, which can be activated by O_2_^−^ from either side of the plasmalemma. Pharmacological activators (↑) and inhibitors (↓) used to study possible players in the ET-1 and Ang II signaling are listed on the sides of the scheme.

Major vascular O_2_^−^ sources include [1,3,9]: (i) mitochondrial respiratory chain and uncoupled NO synthase, which are intracellular systems; (ii) NADPH oxidases (Nox), which are expressed mostly intracellularly; however, isoforms Nox1 and Nox2 are partially plasmalemma-bound enzymes releasing O_2_^−^ intra- and extracellularly [14]; and (iii) xanthine oxidase (XO) which is expressed at a luminal surface of the endothelium. Superoxide that directly inactivates NO and induces endothelial dysfunction (which has been established a predictor of cardiovascular events in patients with coronary artery disease [15]) appears to be extracellular because the dysfunction was reduced by unmodified exogenous superoxide dismutase (SOD) [1,10,16]. Although O_2_^−^ is a short-lived species acting primarily at sites of its generation, all intra- and extracellular O_2_^−^ sources have been implicated in the mechanism of endothelial dysfunction and cytosolic MAPKs activation [1,3,9,17]. Moreover, extracellular ROS has been shown to trigger MAPKs phosphorylation in various systems [17,18,19], suggesting a cross-talk between the intra- and extracellular O_2_^−^ sources and an important regulatory role of extracellular O_2_^−^. Various, albeit incompletely understood, interactions between the O_2_^−^ sources have been previously described, including Nox-mitochondria [9,20,21], Nox-NO synthase [22], and Nox-XO interactions [23]. The present study, which utilizes the isolated guinea-pig heart model [10], aimed to investigate the role of cross-talk between the vascular O_2_^−^ sources in acute ET-1 and AT-II signaling to mediate O_2_^−^ production and endothelial dysfunction, and MAPKs phosphorylation (*i.e.*, intraluminal and cytosolic manifestations of the oxidative stress, respectively). Both agonists were studied because they may differentially activate MAPKs [24]. The detailed hypotheses tested in this study were those outlined in Scheme 1, and their rationales were as follows.

A classic view of AT-II- and ET-1-mediated vascular O_2_^−^ generation revolves around its activation of Nox via receptor-PKC-mediated phosphorylation of p47phox, a regulatory subunit of Nox1 and Nox2, and after longer stimulation via its up regulation of Nox subunit expression [2,25,26,27,28,29]. However, agonists may trigger preconditioning in which the mechanism is thought to involve receptor-PKC-mediated opening of mitochondrial ATP-dependent K^+^ channel (mK_ATP_), mK_ATP_-mediated mitochondrial ROS generation, and further activation of PKC and other target kinases by mitochondrial ROS [30,31]. Thus, these agonists may be expected to coactivate Nox and mitochondria.

While some authors concluded that Nox-derived ROS stimulate mitochondrial ROS [32,33], the others have proposed an upstream role of mitochondria-derived ROS in the activation of Nox in various experimental settings [10,34,35,36,37]. This mitochondrial activator ROS has been proposed to derive from the activation of mK_ATP_ [10,33,38], complex II [10], a reverse electron transfer from complex II to complex I [38], and permeability transition pore [32]. These data led to the proposal that Nox and mitochondria regulate cellular ROS via a positive feedback mechanism [20,21]. However, PKC may act downstream from mitochondria- and Nox-derived ROS [30,31,37,39]. Thus, we aimed to investigate whether PKC is instrumental in the mechanism of Nox-mitochondria cross-talk. Accordingly, we verified that the oxidative stress induced by agonists was similarly prevented by blocking either Nox or mitochondria (specifically mK_ATP_ and complex II), or was similarly mimicked by direct activation of either Nox (NADH) or mitochondria (diazoxide, 3-NPA), and that chelerythrine blocks the effects of Nox and mitochondrial activators.

Nox is known to maintain XO via oxidative mechanism [10,16,23]. However, ROS may activate Nox itself [40]. We hypothesized that Nox-XO interactions are bidirectional and therefore are instrumental in maintaining the intraluminal O_2_^−^, and mediating the regulation of cytosolic ROS and MAPKs by intraluminal O_2_^−^. Thus, we demonstrated that the oxidative stress induced by agonists was similarly prevented by blocking either Nox or XO, that the blockade or direct activation of either of these oxidases resulted in a reciprocal change in the other, and that blocking (SOD, XO inhibition) or triggering intraluminal O_2_^−^ (hypoxanthine activation of XO) prevents or mimics the effects of agonists, respectively.

The present study relies partially on pharmacological tools, which may have off-target effects. Therefore, each molecular target of interest has been examined by means of multiple tools differing in their pharmacological properties (e.g., an inhibitor *vs.* activator) or chemical structure (e.g., 5-HD *vs.* glibenclamide) (Scheme 1). The effects of these various tools appeared to be consistent, increasing the conclusiveness of our results.

## 2. Results and Discussion

### 2.1. O_2_^−^ Generation by ET-1 and AT-II and ET-1-Dependent Effects of AT-II

**Figure 1:**
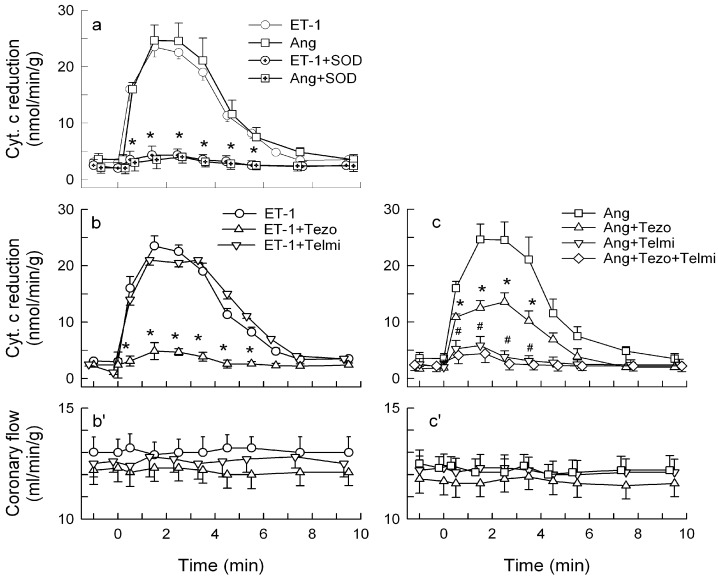
Outflow of reduced cytochrome c (**a–c**) and coronary flow (**b'–c'**) in guinea-pig hearts stimulated with endothelin-1 (ET-1, 25 pM) and angiotensin II (Ang, 20 nM). (**a**) ET-1 and Ang given either alone or in combination with 150 IU/mL SOD; (**b–b'**) ET-1 alone and combined with 5 nM tezosentan or 7.5 μM telmisartan; (**c–c'**) Ang alone, combined with tezosentan, telmisartan or tezosentan + telmisartan. Values are means ± SEM of at least five experiments. * *p* < 0.05 *vs.* ET-1 and Ang group, respectively; ^#^ *p* < 0.05 *vs.* Ang + Tezo group.

The concentration of ET-1 and AT-II was selected in preliminary studies to induce cardiac O_2_^−^ production, which would be similar to that induced by ischemia/reperfusion in our guinea pig heart model, a production that appeared to be mediated by ET-1 [10]. Thus, the ET-1 concentration and equipotent concentration of the other inducers was selected to trigger “physiologically” relevant O_2_^−^ production (see *Section 3.2*). Indeed, at the study concentration, ET-1 (25 pM) and AT-II (20 nM) caused a similar transient outflow of reduced cytochrome c (peaking at 2–3 min and declining to baseline by ~10 min), and this outflow was prevented by SOD (Figure 1a), suggesting that it is mediated by O_2_^−^.

**Figure 2:**
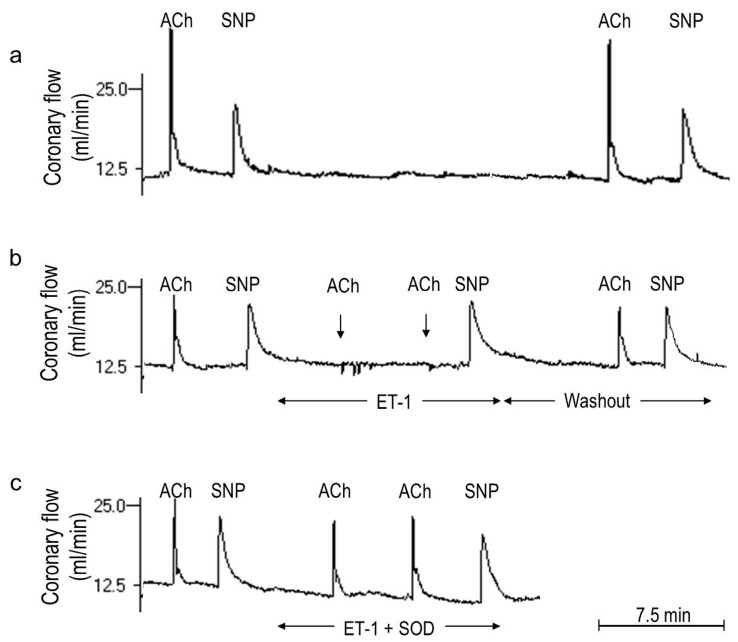
Endothelin-1 (ET-1)-mediated endothelial dysfunction and its prevention by superoxide dismutase (SOD). Shown are three continuous recordings of coronary flow in the Langendorff-perfused guinea pig heart demonstrating responses to boluses of acetylcholine (ACh) and sodium nitroprusside (SNP) in the heart subjected to: (**a**) 35-min control perfusion; ACh and SNP responses obtained at the start and at the end of the perfusion remained unchanged; (**b**) 15-min perfusion with ET-1 (25 pM); ACh and SNP tests were performed before ET-1, in its presence, and after its washout. The response to ACh was completely abolished (see tests at ~5 and ~10 min) and that to SNP remained unaffected in the presence of ET-1, indicating ET-1-mediated endothelial dysfunction. ET-1-washout resulted in an almost complete recovery of the ACh response; (**c**) 15-min of a combined treatment with ET-1 (25 pM) plus SOD (150 IU/mL); SOD completely prevented ET-1-mediated deterioration of the ACh response.

In addition, the hearts treated with the agonists had unchanged vasodilator response to sodium nitroprusside (SNP) and abolished vasodilator response to acetylcholine (ACh), denoting endothelial dysfunction. Also, this latter effect was prevented by SOD (Figure 2). Cytochrome c and SOD are large molecules that scarcely penetrate the plasmalemma, indicating that it was the intraluminal O_2_^−^ that we have measured and that directly inactivated NO to mediate endothelial dysfunction. This supported an endothelial origin of this O_2_^−^, given a short biological O_2_^−^ half-life, its poor tissue diffusibility, and presumably preserved endothelial integrity in the isolated heart preparation. One of the focuses of this study has been on endothelial dysfunction and this preserved endothelial integrity is an apparent advantage of our whole heart model. However, the fact that we have studied whole tissue instead of an endothelial cell line is a limitation of the study as well. In this context, ET-1 and AT-II are known to trigger O_2_^−^ in endothelial cells [27,33]. The agonist also triggered O_2_^−^ in VSMCs [24,25,41] and cardiomyocytes [42,43], and these effects most likely escaped detection in our model. Nevertheless, we believe that O_2_^−^ overproduction in these cell types is associated with similar biochemical changes that can be measured in cardiac homogenates.

Tezosentan, but not telmisartan, inhibited O_2_^−^ generation by ET-1 (Figure 1b). Telmisartan abrogated and tezosentan inhibited the O_2_^−^ generation by AT-II by 43%, and telmisartan abrogated the O_2_^−^ in hearts treated with combined AT-II and tezosentan (Figure 1c), indicating that AT-II triggered O_2_^−^ via the AT_1_ receptor, and similarly as in the vasculature and cardiomyocytes [43,44] using a mechanism that is partially dependent on ET-1 [43]. Hereafter, the hearts were treated with AT-II+tezosentan (Ang-T) to study the ET-1-independent effects of AT-II.

Although the agonists mediated various SOD-inhibitable effects, they did not affect coronary flow (Figure 1b',c'). Likewise, AT-II, at a nonvasomotor level, was reported to trigger O_2_^−^ and endothelial dysfunction in porcine coronary arterioles [45], suggesting that some O_2_^−^-mediated effects, rather than vasomotion, are the agonists’ primary physiological response.

### 2.2. Players in the Signaling by ET-1 and Ang-T to Trigger O_2_^−^, Endothelial Dysfunction, and MAPKs Phosphorylation

**Figure 3:**
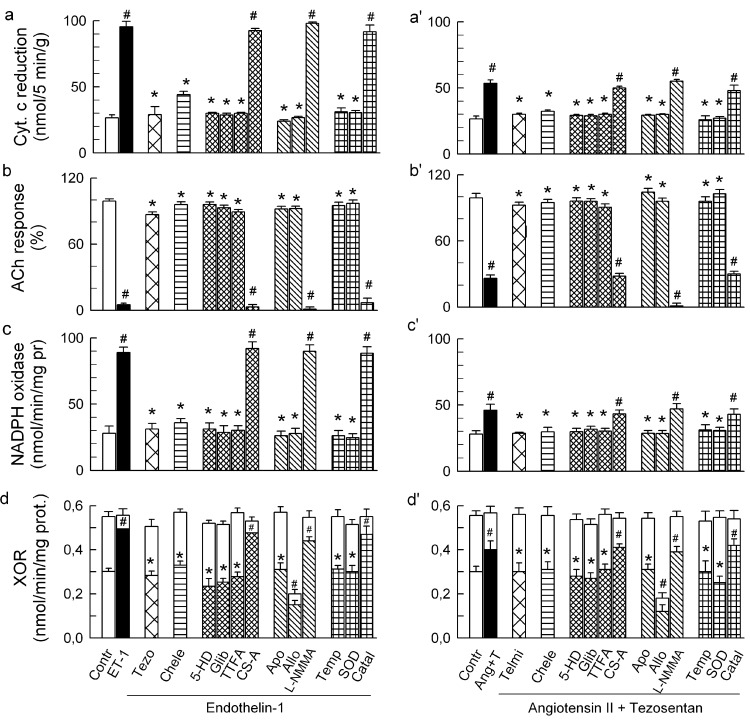
Effect of endothelin-1 (ET-1, 25 pM) (**a–d**) and angiotensin II (20 nM) combined with tezosentan (5 nM) (Ang-T, **a'–d'**) on: total outflow of reduced cytochrome c (as measured during the initial 5 min of the treatment) (**a**,**a'**); normalised acetylcholine (ACh) response (**b**,**b'**); NADPH oxidase activity (studied by NADPH-stimulated cytochrome c reduction) (**c**,**c'**); and xanthine oxidoreductase (XOR) activity (open and hatched parts of the columns represent xanthine dehydrogenase and xanthine oxidase proportion in the total XOR) (**d**,**d'**) in guinea-pig hearts. ET-1 and Ang-T were administered either alone or together with (from left to right): 5 nM tezosentan (Tezo) or 7.5 μM telmisartan (Telmi), 4 μM chelerythrine (Chele), 100 μM 5HD, 3 μM glibenclamide (Glib), 5 μM TTFA, 1 μM cyclosporine A (CS-A), 1 mM apocynin (Apo), 250 μM allopurinol (Allo), 50 μM L-NMMA, 2.5 μM tempol (Temp), 150 IU/mL SOD, and 600 U/mL catalase (Catal). Values are means ± SEM of at least 6 experiments. ^#^ *p* < 0.05 *vs.* control; * *p* < 0.05 *vs.* ET-1 and Ang-T group, respectively.

**Figure 4:**
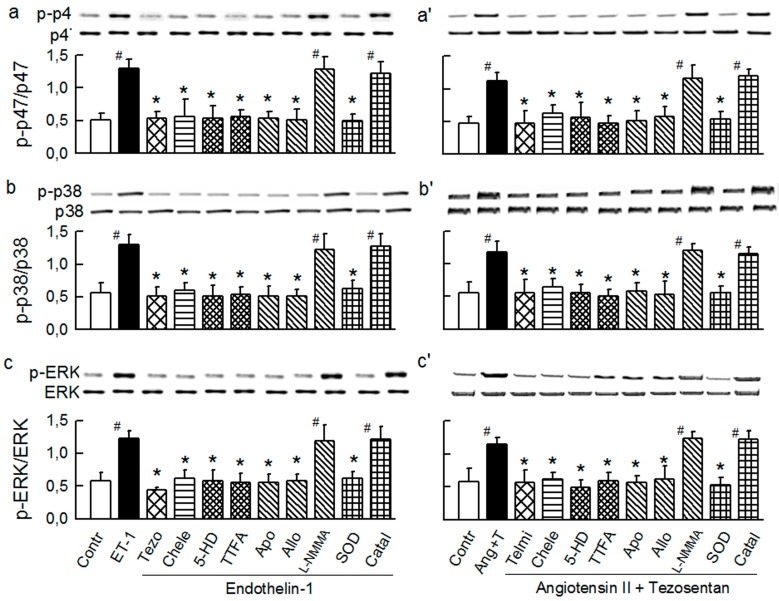
Western blots and densitometry and ratios of phosphorylated and non-phosphorylated proteins: Ser345p47^phox^ (**a–a'**); p38 MAPK (**b–b'**); and ERK1/2 MAPK (**c–c'**) in guinea-pig hearts stimulated with endothelin-1 (ET-1, 25 pM, **a–c**) and angiotensin II (20 nM) combined with tezosentan (5 nM) (Ang-T, **a'–c'**). ET-1 and Ang-T were studied either alone or in combination with (from left to right): 5 nM tezosentan (tezo) or 7.5 μM telmisartan (Telmi), 4 μM chelerythrine (Chele), 100 μM 5HD, 5 μM TTFA, 1 mM apocynin (Apo), 250 μM allopurinol (Allo), 50 μM L-NMMA, 150 IU/mL SOD, and 600 U/mL catalase (Catal). Values are means ± SEM of four experiments. ^#^ *p* < 0.05 *vs.* control; * *p* < 0.05 *vs.* ET-1 and Ang-T group, respectively.

**Figure 5:**
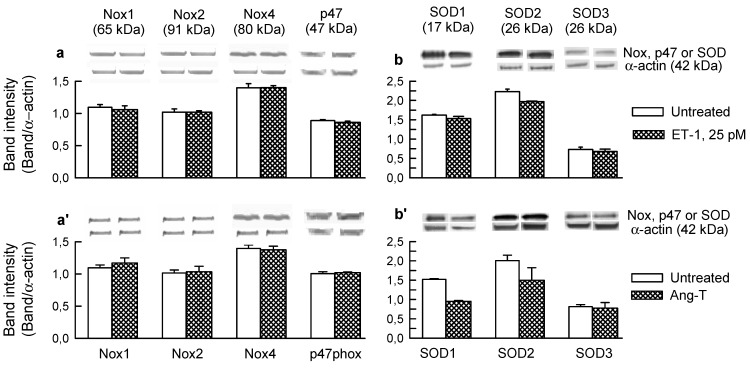
Expression of Nox1, Nox2, Nox4, and p47phox proteins (**a**,**a'**); and of SOD1, SOD2, and SOD3 proteins (**b**,**b'**) in control guinea-pig hearts and in hearts perfused for 10 min with: (**a**,**b**) endothelin-1 (ET-1, 25 pM) and (**a'**,**b'**) 20 nM angiotensin combined with 5 nM tezosentan (Ang-T). In each section, top panel depicts original Western blot of a given protein and of α-actin. The plots below represent the band intensities evaluated using densitometry and normalised against α-actin and are the mean ± SEM of four experiments.

Quantitatively, ET-1 and Ang-T caused ~4-fold and ~2-fold increase in the total O_2_^−^ generation, respectively (Figure 3a,a'). The ET-1 and Ang-T groups had also: (i) abolished the ACh response (Figure 3b,b') and unchanged the SNP response (not shown); (ii) increased Nox activity (Figure 3c,c'), the effect associated with an increased phosphorylation of Ser345-p47phox (Figure 4a,a') and Ser370-p47phox (not shown), but an unchanged expression of p47phox, Nox1, Nox2, and Nox4 proteins (Figure 5a,a'); (iii) increased activity of XO, *i.e.*, the activity of the total xanthine oxidoreductase (XOR) remained unchanged, yet its proportion was largely present in the form of XO (56% ± 3% in controls *vs.* 76% ± 4% and 70% ± 4% in ET-1 and Ang-T groups, respectively, *p* < 0.05), suggesting an increased conversion of xanthine dehydrogenase to XO (Figure 3d,d'); (iv) increased phosphorylation of p38 (Figure 4b,b') and ERK1/2 MAPKs (Figure 4c,c'); (v) neither ET-1 nor Ang-T affected the expression of SOD1, SOD2, and SOD3 proteins (Figure 5b,b').

**Figure 6:**
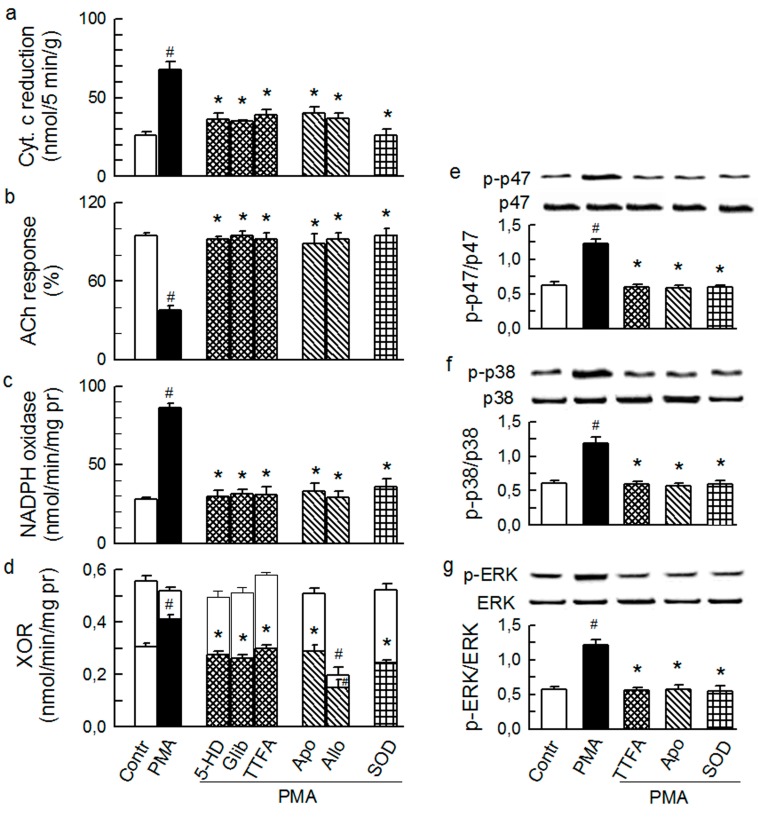
Effect of phorbol 12-myristate 13 acetate (PMA, 1 nM) on the total outflow of reduced cytochrome c (**a**); normalised acetylcholine (ACh) response (**b**); NADPH oxidase activity (**c**); xanthine oxidoreductase activity (XOR) (**d**); phosphorylation of Ser345-p47phox (**e**), p38 (**f**) and ERK1/2 (**g**) in guinea-pig hearts. PMA was given either alone or in combination with (from left to right): 4 μM chelerythrine (Chele), 100 μM 5HD, 3 μM glibenclamide (Glib), 5 μM TTFA, 1 mM apocynin (Apo), 250 μM allopurinol (Allo), and 150 IU/mL SOD. Values are means ± SEM of at least five experiments in **a–d** and of four experiments in **e–g**. # *p* < 0.05 *vs.* control; * *p* < 0.05 *vs.* PMA.

All of the changes by ET-1 and Ang-T were prevented by tezosentan and telmisartan, respectively, and by chelerythrine (Figure 3 and Figure 4) and were mimicked by PMA (Figure 6), suggesting a classical receptor-PKC-dependent mechanism.

The changes by ET-1 and Ang-T (including p47phox/Nox, MAPKs, and XO activation) were prevented by the mitochondrial inhibitors 5-HD, glibenclamide, and TTFA and were insensitive to cyclosporine A (Figure 3 and Figure 4), suggesting a mK_ATP_- and complex II-dependent and mPTP-independent mechanism and that mitochondria are upstream from p47phox/Nox, MAPKs, and XO.

In addition, apocynin and allopurinol but not L-NMMA prevented the changes by ET-1 and Ang-T (Figure 3 and Figure 4), indicating a Nox- and XO-dependent, and NO synthase-independent mechanism. Apocynin had no effect in the untreated hearts (not shown), and it prevented ET-1- and Ang-T-induced activation of p47phox/Nox, MAPKs and XO, implicating MAPKs and XO location downstream from Nox. Allopurinol inhibited XOR and XO activity by ~60% and had no effect on p47phox/Nox and MAPKs in control hearts (not shown). Consequently, XOR was inhibited by ~60% in the hearts perfused with the agonist combined with allopurinol (Figure 2). Allopurinol prevented all of the changes by ET-1 and Ang-T, including the activation of XO, p47phox/Nox and MAPKs (Figure 2 and Figure 3) suggesting that p47phox/Nox and MAPKs were downstream from XO.

Finally, tempol, and SOD, but not catalase and L-NMMA, prevented the effects via ET-1 and Ang-T (Figure 2 and Figure 3), suggesting the role of intraluminal O_2_^−^ in mediating intraluminal (endothelial dysfunction, XO activation) and cytosolic effects (p47phox/Nox, MAPKs activation) by agonists.

The inhibitors that were effective in preventing the effects by agonists were also effective in preventing the effects by PMA (Figure 6).

Altogether, these results suggested that the signaling pathways by ET-1 and Ang-T required PKC, mitochondria (complex II and mK_ATP_), Nox, XO, and the intraluminal O_2_^−^ and that their architecture was nonlinear. The results suggested also that the increased activation of the oxidative enzymes (Figure 3) rather than their increased expression (Figure 5a,a') underlay the agonist-mediated O_2_^−^ generation. Nox activation by the agonists exhibits an acute phase involving PKC-mediated p47phox/Nox activation [25,41,46,47], and a delayed phase involving ERK1/2-dependent [47] upregulation of Nox subunit expression [25,27,28,48]. Here, ET-1 and Ang-T activated p47phox/Nox and ERK1/2. However, the abundance of p47phox, gp91phox (Nox2), as well as Nox1 and Nox 4 protein remained unchanged, suggesting the presence of an acute phase of Nox activation. Persisting oxidative stress may downregulate the expression of SOD isoforms [48]. Here, the abundance of SOD1, SOD2, and SOD3 protein remained unaffected (Figure 5b,b'), supporting the notion that O_2_^−^ overproduction, but not compromised O_2_^−^ elimination, underlay the agonists-mediated acute oxidative stress.

The effects by ET-1 and Ang-T were blocked by their membrane receptor antagonists and chelerythrine and were mimicked by PMA. Moreover, TTFA, 5-HD, apocynin, and allopurinol blocked the effects by agonists and PMA. A simple interpretation of these data is that the agonists triggered oxidative stress via the classic receptor-PKC activation mechanism and that mitochondria, Nox and XO are downstream from the receptor-PKC system. The role of the receptor-PKC system in the activation of XO is likely indirect, given the extracellular location of XO. The mechanism of mitochondrial activation has not been studied here but is likely that mediated by ischemic preconditioning, *i.e.*, receptor-PKC system-mediated phosphorylation and activation of mK_ATP_, resulting in mitochondrial ROS production [31]. In the case of Nox, ET-1, Ang-T, and PMA resulted in apocynin-inhibitable p47phox/Nox activation, suggesting p47phox-dependent Nox activation (apocynin is thought to block p47phox phosphorylation). This suggests Nox1 and/or Nox2 activation as they are the only vascular p47phox-dependent Nox isoforms present [9,49]. Agonists and PMA mediated chelerythrine-inhibitable p47phox phosphorylation at serines 345 and 370, suggesting a PKC-dependent mechanism of phosphorylation. PKC could have acted directly or via some intermediary serine/threonine kinase [50], the possibility not studied here.

### 2.3. Mitochondria-Nox Cross-Talk and Its Reliance on PKC and Intraluminal O_2_^−^

Subsequent experiments were aimed at studying alleged Nox-mitochondria feedback interactions [20,21], and a role of PKC in this mechanism. We verified that the oxidative stress induced by agonists was similarly mimicked by direct activation of either Nox (using NADH) or mitochondria, and that chelerythrine opposes this activation. Mitochondria were stimulated by the mK_ATP_ opener diazoxide and succinate dehydrogenase inhibitor and complex II activator 3-NPA. Both these interventions are known to trigger mitochondrial ROS [31,51,52].

**Figure 7:**
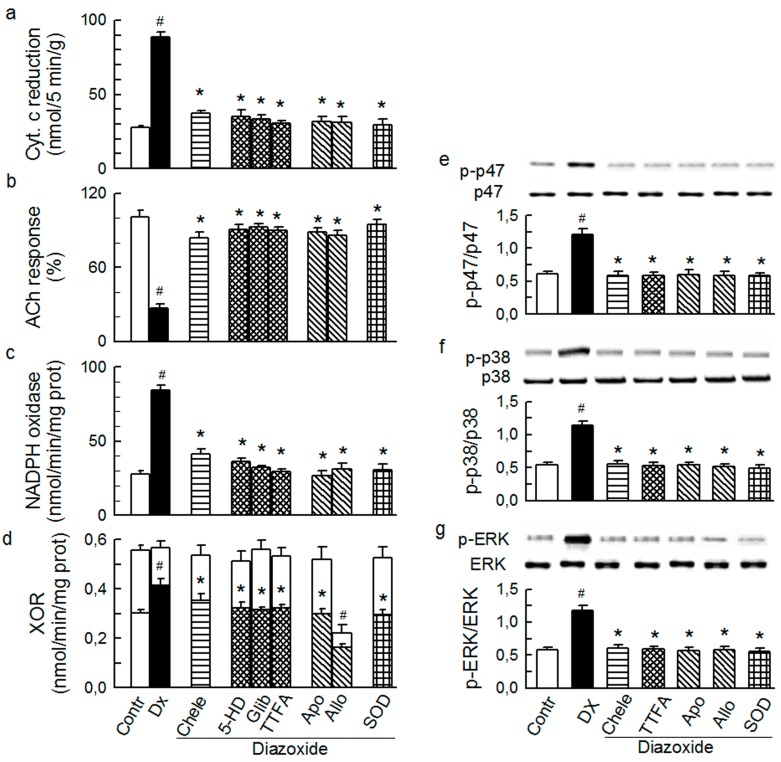
Effect of diazoxide (Dx, 2.5 μM) on the total outflow of reduced cytochrome c (**a**); normalised acetylcholine (ACh) response (**b**); NADPH oxidase activity (**c**); xanthine oxidoreductase activity (XOR) (**d**); the phosphorylation of Ser345-p47phox (**e**), p38 (**f**) and ERK1/2 (**g**) in guinea-pig hearts. Diazoxide was administered either alone or in combination with (from left to right): 4 μM chelerythrine (Chele), 100 μM 5HD, 3 μM glibenclamide (Glib), 5 μM TTFA, 1 mM apocynin (Apo), 250 μM allopurinol (Allo), and 150 IU/mL SOD. Values are means ± SEM of at least five experiments in **a–d** and of four experiments in **e–g**. ^#^ *p* < 0.05 *vs.* control; * *p* < 0.05 *vs.* Dx.

**Figure 8:**
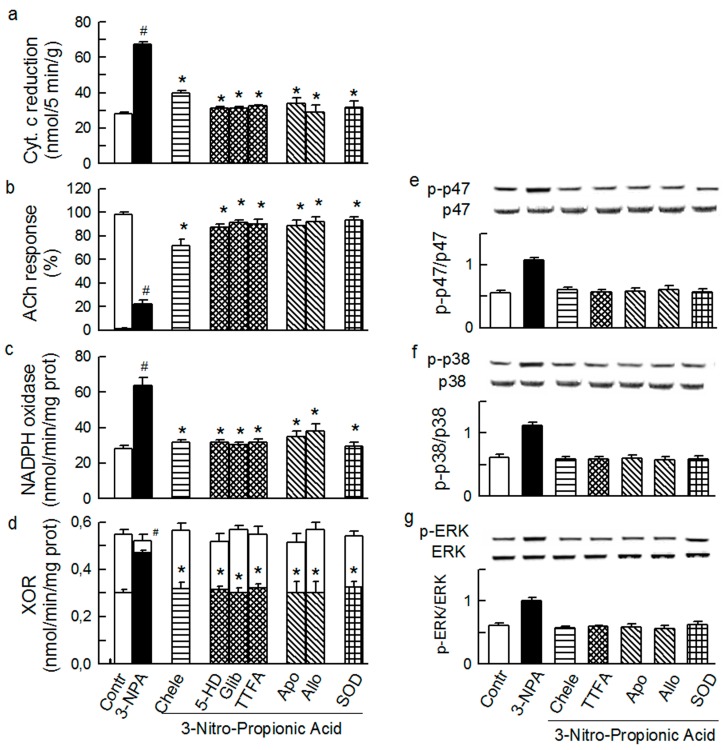
Effect of 3-Nitro-Propionic Acid (3-NPA, 5 mM) on the total outflow of reduced cytochrome c (**a**); normalised acetylcholine (ACh) response (**b**); NADPH oxidase activity (**c**); xanthine oxidoreductase activity (XOR) (**d**); phosphorylation of Ser345-p47phox (**e**), p38 (**f**) and ERK1/2 (**g**) in guinea-pig hearts. 3-NPA was administered either alone or in combination with (from left to right): 4 μM chelerythrine (Chele), 100 μM 5HD, 3 μM glibenclamide (Glib), 5 μM TTFA, 1 mM apocynin (Apo), 250 μM allopurinol (Allo), and 150 IU/mL SOD. Values are means ± SEM of at least five experiments in **a–d** and of four experiments in **e–g**. ^#^ *p* < 0.05 *vs.* control; * *p* < 0.05 *vs.* 3-NPA.

**Figure 9:**
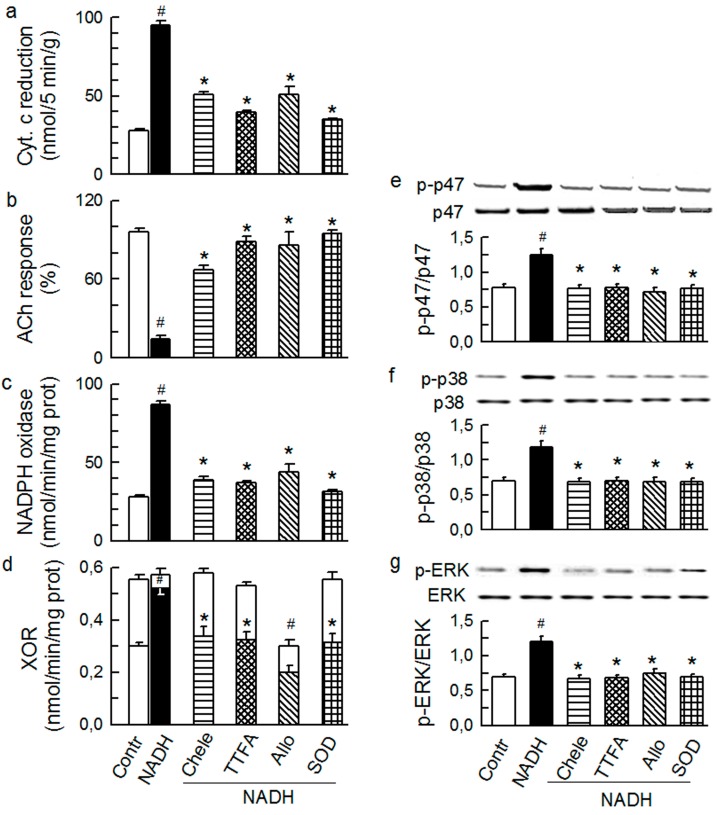
Effect of NADH (2.5 μM) on the total outflow of reduced cytochrome c (**a**); normalised acetylcholine (ACh) response (**b**); NADPH oxidase activity (**c**); xanthine oxidoreductase activity (XOR) (**d**); the phosphorylation of Ser345-p47phox (**e**), p38 (**f**) and ERK1/2 (**g**) in guinea-pig hearts. NADH was administered either alone or in combination with (from left to right): 4 μM chelerythrine (Chele), 5 μM TTFA, 250 μM allopurinol (Allo), and 150 IU/mL SOD. Values are means ± SEM of at least five experiments in **a–d** and of four experiments in **e–g**. ^#^ *p* < 0.05 *vs.* control; * *p* < 0.05 *vs.* NADH.

Diazoxide (2.5 μM), 3-NPA (5 mM), and NADH (2.5 mM) caused a transient burst in O_2_^−^ outflow (*Section 3.2*), which was equipotent to that triggered by ET-1 and AT-II in this study. In addition, diazoxide (Figure 7), 3-NPA (Figure 8), and NADH (Figure 9) mediated increased total O_2_^−^ generation, depressed ACh response, increased activity of Nox and XO, and increased phosphorylation of p47phox and MAPKs.

Apocynin and chelerythrine abolished the effects by mitochondrial activators diazoxide and 3-NPA (including p47phox/Nox, MAPKs, and XO activation, Figure 7 and Figure 8), indicating that p47phox/Nox, PKC, and XO were downstream from mitochondria. Furthermore, the effects by the putative Nox activator NADH (which indeed mediated p47phox/Nox activation and typical oxidative stress) were abolished by mitochondrial inhibitors 5-HD and TTFA, and by chelerythrine (Figure 9), suggesting that mitochondria, PKC, and XO were downstream from p47phox/Nox. These data are consistent with the hypothesis that (i) Nox and mitochondria mutually maintain their activity via a positive feedback mechanism [20,21]; (ii) PKC is instrumental in this mechanism; and (iii) the activity of MAPKs and XO is dependent on mitochondria, Nox, and PKC working as a functional complex.

Interestingly, while TTFA and chelerythrine inhibited the effects by diazoxide (used in a small 2.5 μM concentration that is thought to not inhibit succinate dehydrogenase) (Figure 7), 5-HD, glibenclamide, and chelerythrine blocked the effect by 3-NPA (Figure 8), suggesting that mK_ATP_ and complex II mutually control their activities, and also via PKC.

Finally, all of the intraluminal (O_2_^−^ production, endothelial dysfunction, XO activation) and cytosolic effects (p47phox/Nox and MAPKs activation) by diazoxide, 3-NPA and NADH (Figure 7, Figure 8 and Figure 9) were opposed by allopurinol and SOD, the interventions presumably acting intraluminally. These results, and those cited earlier, implicate a role of an intraluminal O_2_^−^ in the mechanism of XO activation by the agonists and other studied O_2_^−^-inducers, and also an obligatory role of XO and/or the intraluminal O_2_^−^ in maintaining p47phox/Nox and MAPKs activity.

The mechanism of PKC activation by Nox and mitochondria has not been studied here, but it was likely ROS-dependent. First, PKC may be activated by ROS via direct oxidative modification or indirectly via a modification of associated molecular redox-sensors, e.g., c-Src non-receptor tyrosine kinase [50]. Indeed, c-Src is shown to serve as an upstream and downstream modulator of Nox in the signaling cascade by AT-II and ET-1 [25,41,47,51], and an upstream regulator of MAPKs in other systems [47,50]. Second, ROS from mitochondria [30,31] and Nox [39] have been reported to activate PKC (specifically PKCε) in various experimental settings. Furthermore, a hypoxia-induced increase in mitochondrial ROS generation has been shown to activate Nox via PKCε in pulmonary artery smooth muscle cells [37]. We speculate that agonists, acting via the receptor-PKC activation mechanism, trigger an initial O_2_^−^ generation by mitochondria and Nox and that this O_2_^−^ mediates further activation of PKC (the same or its other pool), which subsequently amplifies the cytoplasmic O_2_^−^ generation by the mitochondria and Nox, but also supports an extracellular transmission of the oxidative signal that is seen in our model as intraluminal O_2_^−^ production, XO activation, and endothelial dysfunction (*c.f.* Scheme 1).

Indeed, increased conversion of xanthine dehydrogenase to XO, as seen in our model, may be mediated by irreversible proteolytic conversion or by reversible oxidation of thiol groups [53]. Here, activation of XO was prevented by SOD (but not by catalase and L-NMMA, Figure 3), implicating its intraluminal O_2_^−^-dependent and an intraluminal H_2_O_2_-, NO- and peroxynitrite-independent mechanism. We speculate that the cytosolic PKC-mitochondria-Nox system was the source of this activating O_2_^−^. The cytosolic O_2_^−^ could enter the extracellular space through diffusion via the membrane anion channels. Alternatively, the O_2_^−^ could be released extracellularly by the plasmalemma-bound fraction of Nox1/Nox2, which may release O_2_^−^ intra- and extracellularly [14], and which is a constituent of the PKC-mitochondria-Nox system. Nox has been previously suggested to maintain XO via the oxidative mechanism in various experimental settings [10,16,23]. Our data suggest an instrumental role of the PKC-mitochondria-Nox system in this mechanism in our model.

### 2.4. Cross-Talk between Cytoplasmic and Intraluminal Pool of ROS

Subsequent experiments were aimed at further testing the hypothesis that Nox-XO interactions are bidirectional and therefore are instrumental in maintaining the intraluminal O_2_^−^ as well as the activity of the cytosolic PKC-mitochondria-Nox system and MAPKs. Thus, we verified that the direct Nox activation with NADH results in the activation of XO, the direct XO activation with hypoxanthine results in the activation of Nox, and that the manifestations of oxidative stress by NADH and hypoxanthine are similar and are blocked by the same set of the inhibitors.

**Figure 10:**
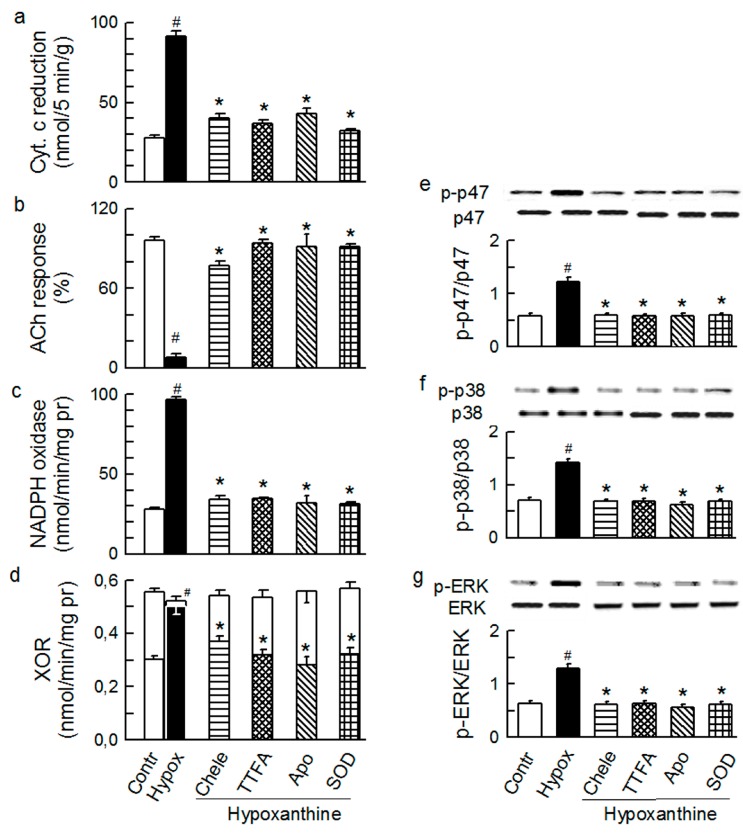
Effect of hypoxanthine (20 μM) on the total outflow of reduced cytochrome c (**a**); normalised acetylcholine (ACh) response (**b**); NADPH oxidase activity (**c**); xanthine oxidoreductase activity (XOR) (**d**); phosphorylation of Ser345-p47phox (**e**), p38 (**f**) and ERK1/2 (**g**) in guinea-pig hearts. Hypoxanthine was administered either alone or in combination with (from left to right): 4 μM chelerythrine (Chele), 5 μM TTFA, 1 mM apocynin (Apo), and 150 IU/mL SOD. Values are means ± SEM of at least five experiments in **a–d** and of four experiments in **e–g**. ^#^ *p* < 0.05 *vs.* control; * *p* < 0.05 *vs.* NADH.

Indeed, NADH (2.5 mM, Figure 9) and hypoxanthine (20 μM, Figure 10) resulted in similarly increased total O_2_^−^ generation, depressed ACh response, increased enzymatic activity of Nox and XO, and increased phosphorylation of p47phox and MAPKs. The effects by NADH (including p47phox/Nox and MAPKs activation), were abolished by allopurinol and those by hypoxanthine (including p47phox/Nox and MAPKs activation) were abolished by apocynin. In addition, the effects by NADH and hypoxanthine were abolished by the cytosolic inhibitors chelerythrine, TTFA, and 5-HD, and by intraluminally acting SOD.

Thus, we demonstrate that various O_2_^−^-inducers acting intracellularly (ET-1, Ang-T, PMA, diazoxide, 3-NPA, NADH), trigger extracellular effects (XO activation, intraluminal O_2_^−^ generation, endothelial dysfunction) that are inhabitable by interventions thought to act intracellularly (chelerythrine, TTFA, 5-HD, apocynin). These data suggested that the intraluminal manifestations of the oxidative stress are mediated by the cytosolic PKC-mitochondria-Nox system. Conversely, putative intraluminal inhibitors SOD and allopurinol (but not catalase and L-NMMA) abolished the cytosolic effects by ET-1, Ang-T, PMA, diazoxide, 3-NPA, and NADH (*i.e.*, p47phox/Nox and MAPKs activation). In addition, XO activator hypoxanthine mediated p47phox/Nox and MAPKs activation, which was abolished by the cytoplasmic inhibitors (chelerythrine, TTFA, and apocynin), but also by SOD. Thus, changes in the intraluminal O_2_^−^ (but not in H_2_O_2_, NO and peroxynitrite) are translated into reciprocal changes in PKC-mitochondria-Nox system activity. This points to the cellular outside-in O_2_^−^ signaling as an indispensable element of the oxidative signaling pathway by agonists and other tested O_2_^−^-inducers. The electrochemical driving force for negatively charged O_2_^−^ is expected to favor rather its outward movement. Because Nox can be directly activated by ROS [40], we speculated that it was plasmalemma-bound fraction of Nox1/2 that was activated by the intraluminal O_2_^−^, resulting in the intra- and extracellular O_2_^−^ release by the enzyme. This initial cytoplasmic O_2_^−^ release would then undergo amplification by the mechanism involving O_2_^−^-dependent PKC activation and further PKC-dependent activation of mitochondria- and p47phox/Nox-derived cytoplasmic O_2_^−^ release. Nox1/2, the alleged constituent of the PKC-mitochondria-Nox system, would release increased amounts of O_2_^−^ extracellularly/intraluminally (Scheme 1). Consistent with this hypothesis, hypoxanthine and NADH mediate chelerythrine- and TTFA-inhibitable p47phox (as well as MAPKs) phosphorylation, and XO and Nox activation. We speculated that hypoxanthine, acting via XO-derived O_2_^−^, and NADH, acting directly on Nox, stimulated a small initial O_2_^−^ release by Nox, which resulted in PKC-mediated phosphorylation and subsequently in activation of the PKC-mitochondria-Nox system.

In summary, the effects by the extracellular activator hypoxanthine required the activation of the cytoplasmic PKC-mitochondria-Nox system, and the effects by cytoplasmic activators (ET-1, Ang-T, diazoxide, 3-NPA, NADH) required the activation of intraluminal XO and O_2_^−^. These data supported the hypothesis that PKC, mitochondria, Nox and XO participate in the studied signaling pathway as one functional complex and that this pathway involves cellular inside-out and outside-in transmission of the oxidative signal.

## 3. Experimental Section

### 3.1. Chemicals

Apocynin and thenoyltrifluoroacetone (TTFA) were purchased from Aldrich, tezosentan was a gift from Dr. Martine Clozel (Actelion, Switzerland), and the other chemicals were obtained from Sigma. Phorbol 12-myristate 13 acetate (PMA) and apocynin were dissolved in DMSO, allopurinol in 96% ethanol, and ET-1, Ang, and tezosentan in perfusion solution containing 0.02% albumin (bovine serum, fatty acid free, Sigma). Previously, we verified that the vehicles used (0.0025% DMSO and 0.034% ethanol) affected neither the endothelial nor contractile function in our perfused guinea-pig heart model [10,54].

### 3.2. Heart Preparation and Protocols

All of the procedures conformed to the *Guide for the Care and Use of Laboratory Animals* (National Institutes of Health (NIH) publication No. 85-23, revised 1996) and were approved by the local Animal Subject Ethics Committee. Guinea-pigs of either sex (260–320 g) were studied in October until April to avoid complications related to the summer-associated cardiac O_2_^−^ overproduction [48].

Details of the perfused guinea-pig heart model have been published elsewhere [10]. The hearts were perfused using the Langendorff method, at a perfusion pressure of 70 mm Hg, with Krebs-Henseleit buffer (KHB) containing 11 mM glucose, and gassed with 95% O_2_ + 5% CO_2_ gas mixture. The hearts were not paced.

The experiments were designed to study the cardiac effects of several agents inducing vascular O_2_^−^ production (“O_2_^−^-inducers”), and whether their effects would be opposed by various “inhibitors”.

The inducers included: ET-1 (25 pM), angiotensin II (20 nM), PKC activator phorbol 12-myristate 13 acetate (PMA, 1 nM), mK_ATP_ opener diazoxide (2.5 μM) [31,51], an irreversible inhibitor of succinate dehydrogenase (the mitochondrial complex II enzyme) 3-nitropropionic acid (3-NPA, 5 mM) [52], and substrates and activators of Nox (β-NADH, 2.5 mM) and of XO (hypoxanthine, 20 μM).

**Figure 11:**
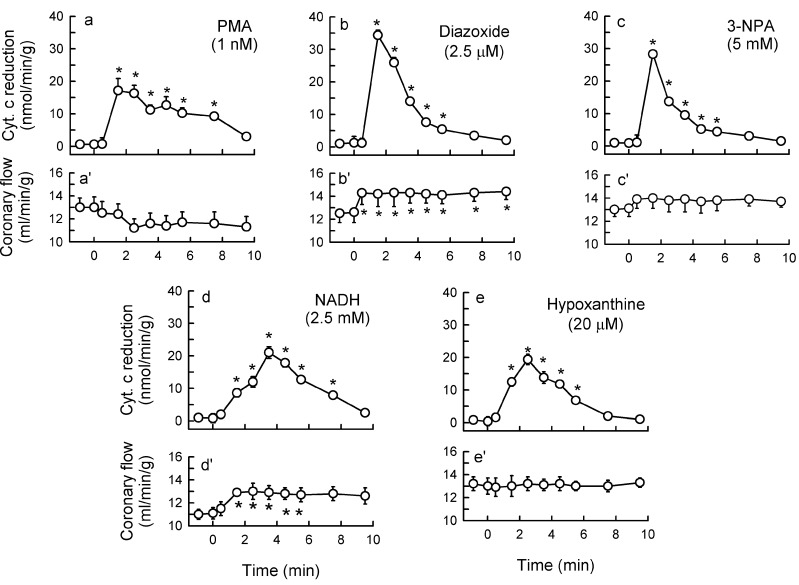
Outflow of reduced cytochrome c (**a–e**) and coronary flow (**a'–e'**) in guinea pig hearts stimulated for 10 min with 1 nM PMA (**a**,**a'**), 2.5 μM diazoxide (**b**,**b'**), 5 mM 3-nitr-propionic acid (3-NPA, **c**,**c'**), 2.5 mM NADH, **d**,**d'**) and 20 μM hypoxanthine (**e**,**e'**). The agents were introduced into the perfusate at time 0. Values are means ± SEM of **5–6** experiments. * *p* < 0.05 *vs.* basal measurements.

The concentration of each inducer was selected in preliminary concentration-response studies to induce cardiac O_2_^−^ production, which would be quantitatively similar to that induced by ischemia/reperfusion in our guinea-pig heart model, a production that appeared to be mediated by ET-1 [10]. The point was to select an ET-1 concentration and equipotent concentration of other inducers (Figure 11) that would trigger “physiologically” relevant O_2_^−^ production.

The inhibitors blocked: endothelin ET_A_/ET_B_ receptors (tezosentan), angiotensin AT_1_ receptor (telmisartan), PKC (chelerythrine), mK_ATP_ (5-hydroxydecanoic acid sodium, 5-HD, and glibenclamide), mitochondrial complex II (thenoyltrifluoroacetone, TTFA), mPTP (cyclosporine A), Nox (apocynin), XO (allopurinol), and NO synthase (L-NMMA). Furthermore, superoxide dismutase (SOD), a cell-permeable SOD-mimetic (tempol), and catalase were studied.

All of the hearts had a 30 min stabilization perfusion and then KHB was supplemented with cytochrome c (*Section 3.4*), and the perfusion was continued for the next 30 min (test perfusion). Four types of experiments were performed: (i) Control—the hearts were only subjected to the test perfusion; (ii) The inhibitors administered alone; they were added to the perfusate at 5 min of the test perfusion and the perfusion continued until the end of the protocol; (iii) The O_2_^−^ inducers administered alone; at 15 min during the test perfusion, ET-1, AT-II, PMA, and diazoxide were infused via a side arm of the aortic cannula (as a constant infusion 1/100 of coronary flow) and the other inducers were added directly to the perfusate; (iv)The O_2_^−^ inducers were administered on top of the inhibitors; the protocol was performed using a combination of protocols 2 and 3.

Coronary flow (Transit Time Flowmeter Module, Hugo Sachs Elektronik, March, Germany) and endothelial function were measured and the effluent was collected for cardiac O_2_^−^. Finally, the hearts were freeze-clamped and stored at −72 °C for biochemistry.

### 3.3. Endothelium-Dependent Vasodilatation

The vasodilator responses to acetylcholine (ACh) and sodium nitroprusside (SNP) served as measures of an agonist-induced endothelium-dependent and endothelium-independent vascular function, respectively [54]. The coronary flow was continuously recorded and the 50 µL bolus of 10 µM ACh or 400 µM SNP was injected into the aortic cannula and a 1 min sample of the effluent was collected, weighed, and a 1 min coronary overflow produced by the drug was calculated (*c.f.* Figure 2). The ACh and SNP responses were studied at the beginning and at the conclusion of the test perfusion in the experimental protocols 1 and 2, and at 10–15 min and at the conclusion of the test perfusion in the protocols 3 and 4. Data from two consecutive tests were used to calculate a normalised vasodilator response to the drug using the formula: a drug-induced 1 min coronary overflow at the conclusion of the protocol/the overflow during the initial test × 100%.

### 3.4. Superoxide

The hearts were perfused with KHB containing 10 µM succinylated ferricytochrome c and 600 U/mL catalase (cytochrome c) [10]. The optical density of the coronary effluent was measured at 550 nm. The cardiac O_2_^−^ formation was calculated using the molar absorbance coefficient for cytochrome c of 21 mM^−1^·cm^−1^.

### 3.5. Western Immunoblot Analyses

The method used has been previously reported [48]. Homogenized frozen ventricles were subjected to SDS-PAGE electrophoresis (4%–20% Precise Protein Gels, Thermo Scientific, Rockford, IL, USA) and electroblotting on nitrocellulose membranes (Whatman Gmbh, Dassel, Germany). The membranes were first incubated with the primary antibodies against proteins listed in Table 1 and then with the appropriate secondary antibodies. Immunodetection was accomplished with SuperSignal West Pico (Thermo Scientific, Rockford, IL, USA). The bands were evaluated by densitometry and normalized either to the α-actin or, in case of phospho-proteins, to their unphosphorylated counterparts.

### 3.6. NADPH and Xanthine Oxidases Activity

The method used has been previously reported [10]. Frozen ventricles were homogenised in phosphate buffer (50 mM, pH 7.4) containing 1 mM EDTA, 10 mM dithiothreitol, and 1 mM phenylmethylsulfonyl fluoride. Half of the homogenate was centrifuged at 800× *g* for 10 min at 4 °C and the other half at 27,000× *g* for 30 min at 4 °C and the resulting supernatants were used for NADPH oxidase (Nox) and xanthine oxidase (XO) assays, respectively.

Nox activity was determined by SOD-inhibitable NADH- and NADPH-mediated cytochrome c reduction assay. Equal protein samples (5 μg, Bradford method) of the supernatant were incubated in 1 mL of 50 mM phosphate buffer (pH 7.4) containing 20 μM succinylated ferricytochrome *c*, 3000 U/mL catalase, and 100 μmol NADH of NADPH for 45 min at 37 °C, and the absorbance was measured at 550 nm. All assays were performed in parallel with and without SOD (500 U/mL).

**Table 1:** Primary and secondary antibodies used in Western blot analyses.

Primary	Host	Dilution	Cat. No.	Company	Secondary	Dilution	Cat. No.	Company
ERK ½	Mouse	1:1000	135900	SCB ^1^	Rabbit anti-mouse AP ^7^	1:500	358915	SCB
p-ERK ½	Rabbit	1:1000	16982	SCB	Mouse anti-rabbit AP	1:500	2358	SCB
p38	Rabbit	1:1000	7149	SCB	Mouse anti-rabbit AP	1:500	2358	SCB
p-p38	Rabbit	1:1000	17852	SCB	Mouse anti-rabbit AP	1:500	2358	SCB
p47^phox^ (total)	Rabbit	1:1000	7660	SCB	Mouse anti-rabbit AP	1:500	2358	SCB
p47^phox^ (unphosphoryl.)	Rabbit	1:1000	4502808	Sigma ^2^	Goat anti-rabbit AP	1:1000	0418	Sigma
p47^phox^ (pSer345)	Rabbit	1:1000	8391	ABC ^3^	Mouse anti-rabbit AP	1:500	2358	SCB
p47^phox^ (pSer370)	Rabbit	1:1000	4504288	Sigma	Goat anti-rabbit AP	1:1000	0418	Sigma
Nox1	Rabbit	1:1000	55831	Abcam ^4^	Goat anti-rabbit AP	1:1000	6722	Abcam
Nox2	Rabbit	1:1000	31092	Abcam	Goat anti-rabbit AP	1:1000	6722	Abcam
Nox4	Rabbit	1:1000	60940	Abcam	Goat anti-rabbit AP	1:1000	6722	Abcam
SOD1	Rabbit	1:1000	NB1001955	NB ^5^	Goat anti-rabbit HRP ^8^	1:5000	P0448	Dako ^9^
SOD2	Rabbit	1:1000	SOD111	AD ^6^	Goat anti-rabbit HRP	1:5000	P0448	Dako
SOD3	Rabbit	1:1500	83108	Abcam	Goat anti-rabbit HRP	1:5000	P0448	Dako
α-Actin	Rabbit	1:1000	15734	Abcam	Mouse anti-rabbit AP	1:500	2358	SCB

^1^ Santa Cruz Biotechnology Inc., Santa Cruz, CA, USA; ^2^ Sigma-Aldrich Manufacturing, LLC, Saint Louis, MO, USA; ^3^ Assay Biotechnology Company Inc., Sunnyvale, CA, USA; ^4^ Abcam, Cambridge, UK; ^5^ Novus Biologicals, Littleton, CO, USA; ^6^ Assay Designs, Ann Arbor, MI, USA; ^7^ AP, Alkaline Phosphatase; ^8^ HRP, Horse-Radish Peroxidase; ^9^ Dako, Glostrup, Denmark.

XO activity was determined by allopurinol- and uricase-inhibitable uric acid formation. The supernatants (1.2 mL) were passed over a Sephadex G-25 column. The column was eluted with the homogenization buffer. To measure XO, the enzyme-rich eluent was incubated in 1 mL of the phosphate buffer containing 50 μM xanthine, 0.13 mM EDTA, and 600 U/mL catalase for 10 min at 37 °C, and the absorbance was measured at 293 nm. The procedure for the determination of the total xanthineoxidoreductase (XOR) was identical to the above, with the exception that 0.6 mM NAD^+^ was added to all cuvettes. All experiments were performed in parallel with and without 250 μM allopurinol or 100 mU/mL uricase. Supernatants from allopurinol-perfused hearts were not passed over the column to prevent loss of the allopurinol into the Sephadex. The recovery of XOR and XO from the supernatant increased by ~21% with the Sephadex treatment. Therefore, their activities in the allopurinol-treated hearts were corrected accordingly. The enzymes activities were expressed in pmol/min/mg protein of urate produced.

### 3.7. Statistics

Data were confirmed for normality and were expressed as the mean ± SEM. The significance of differences among groups was calculated using one-way analysis of variance followed by Bonferroni’s procedure. To test for the differences in the normalised responses to ACh, the Kruskal-Wallis test followed by the Mann-Whitney test was performed. A value of *p* < 0.05 was considered significant.

## 4. Conclusions and Clinical Perspectives

Physiologically relevant concentrations of ET-1 and AT-II mediate oxidative stress, but not vasomotion, in isolated guinea-pig heart. Manifestations of oxidative stress are cytoplasmic (e.g., MAPKs activation) and intraluminal (intraluminal O_2_^−^ production, endothelial dysfunction, XO activation). Although the agonists and other studied O_2_^−^-inducers act via distinct molecular targets (membrane receptors, PKC, complex II, mK_ATP_, Nox, XO), they all seem to trigger the same nonlinear signaling pathway. This pathway consists of cytoplasmic (PKC, Nox, mitochondria) and intraluminal components (XO, intraluminal O_2_^−^) that mutually control their activities and provide that the cytosolic and the intraluminal O_2_^−^-pool remain in equilibrium. The O_2_^−^-inducers mediated also a standard oxidative response, altogether indicating that the mechanism of oxidative stress (at least of its acute phase and in our model) was non-specific.

It is attractive to speculate that a similar universal mechanism underlies oxidative stress mediated by its other known inducers acting via the receptor-PKC system (various hormones and cytokines), mitochondria (e.g., diabetes, nitroglycerin, mutations of succinate dehydrogenase) [36,55,56] and XO (e.g., oxidative stress in ischemia/reperfusion or in exercising skeletal muscle) [57,58]. A practical corollary of the universal mechanism would be that, for the optimal anti-oxidative medication, it may be more important to develop a drug possessing unique physico-chemical properties rather than targeting a specific oxidant enzyme.

Endothelial dysfunction in humans is a systemic phenomenon presumably because it is mediated by risk factors acting systemically. An alternative explanation would be that due to the postulated cross-talk between cytosolic and intravascular pool of ROS (Scheme 1), a locally induced oxidative stress and endothelial dysfunction can “wander” from cell to cell within the vascular wall.

Endothelial dysfunction, especially that mediated by vascular ROS, has been established as a predictor of cardiovascular events in patients with coronary artery disease [15]. The interpretation of this was that ROS *per se*, rather than endothelial dysfunction, directly contributed to the mechanism of coronary artery disease. We demonstrated that intraluminal O_2_^−^, which indeed appeared to mediate endothelial dysfunction, simultaneously mediated MAPKs phosphorylation. Thus, at least in our model, ROS-induced endothelial dysfunction is a marker of the MAPKs activation thought ultimately to mediate vascular remodeling and inflammation [1,2,3,7,8,9,22].
